# Effects of Paracetamol on NOS, COX, and CYP Activity and on Oxidative Stress in Healthy Male Subjects, Rat Hepatocytes, and Recombinant NOS

**DOI:** 10.1155/2014/212576

**Published:** 2014-03-31

**Authors:** Arne Trettin, Anke Böhmer, Maria-Theresia Suchy, Irmelin Probst, Ulrich Staerk, Dirk O. Stichtenoth, Jürgen C. Frölich, Dimitrios Tsikas

**Affiliations:** ^1^Institute of Clinical Pharmacology, Hannover Medical School, 30625 Hannover, Germany; ^2^Department of Biochemistry and Molecular Cell Biology, Georg-August University, 37073 Goettingen, Germany

## Abstract

Paracetamol (acetaminophen) is a widely used analgesic drug. It interacts with various enzyme families including cytochrome P450 (CYP), cyclooxygenase (COX), and nitric oxide synthase (NOS), and this interplay may produce reactive oxygen species (ROS). We investigated the effects of paracetamol on prostacyclin, thromboxane, nitric oxide (NO), and oxidative stress in four male subjects who received a single 3 g oral dose of paracetamol. Thromboxane and prostacyclin synthesis was assessed by measuring their major urinary metabolites 2,3-dinor-thromboxane B_2_ and 2,3-dinor-6-ketoprostaglandin F_1**α**_, respectively. Endothelial NO synthesis was assessed by measuring nitrite in plasma. Urinary 15(*S*)-8-*iso*-prostaglanding F_2**α**_ was measured to assess oxidative stress. Plasma oleic acid oxide (*cis*-EpOA) was measured as a marker of cytochrome P450 activity. Upon paracetamol administration, prostacyclin synthesis was strongly inhibited, while NO synthesis increased and thromboxane synthesis remained almost unchanged. Paracetamol may shift the COX-dependent vasodilatation/vasoconstriction balance at the cost of vasodilatation. This effect may be antagonized by increasing endothelial NO synthesis. High-dosed paracetamol did not increase oxidative stress. At pharmacologically relevant concentrations, paracetamol did not affect NO synthesis/bioavailability by recombinant human endothelial NOS or inducible NOS in rat hepatocytes. We conclude that paracetamol does not increase oxidative stress in humans.

## 1. Introduction

Nitric oxide (NO), prostaglandin (PG) I_2_, that is, prostacyclin (PGI_2_), and thromboxane A_2_ (TxA_2_) are important short-lived signaling molecules involved in many physiological and pathological processes. Thus, PGI_2_ and NO are potent vasodilators and inhibitors of platelet aggregation. Contrarily, TxA_2_ is a strong vasoconstrictor and inductor of platelet aggregation. NO is synthesized from L-arginine (Arg) by constitutive and inducible NO synthase (NOS) isoforms. Prostaglandin H synthase (PGHS) isoforms, generally termed cyclooxygenase (COX), convert arachidonic acid (AA) to the collectively named prostanoids. The L-arginine/NO pathway is generally accepted to interact with the COX pathway and to modulate its activity [1–4]. For instance, the inducible NOS (iNOS) isoform has been shown to bind to the inducible COX isoform (COX-2) and to *S*-nitrosylate and activate COX-2 [2]. The role of NO in prostaglandin biology has been recently updated by Kim [4]. Potential mechanisms of direct NOS-COX cross-talk may include (1) binding of NO to the iron atom of the heme group of COX, (2) reaction of the nitrosyl cation (NO^+^) with sulfhydryl (SH) groups of cysteine (Cys) moieties of COX to form *S*-nitroso-COX, and (3) reaction of peroxynitrite (ONOO^−^), that is, the reaction product of NO radical (^*∙*^NO) and superoxide radical anion (O_2_^∙−^) produced either by NOS itself or by other enzymes including COX and CYP [3], with SH groups of Cys residues or with tyrosine (Tyr) residues of COX being involved in the catalytic process [2]. *S*-Nitrosylation of COX-Cys moieties by higher oxides of NO, notably dinitrogen trioxide (N_2_O_3_), and by ONOO^−^ and *S*-transnitrosylation of COX-Cys moieties by low-molecular-mass *S*-nitrosothiols have been shown to both enhance and inhibit COX activity. Nitration of Tyr residues located in the catalytic domain of COX is assumed to inhibit COX activity [2, 4–6]. On the other hand, ONOO^−^ has been reported to enhance COX activity presumably by increasing the peroxide concentration that is required for the peroxidase activity of COX [7].

Paracetamol (acetaminophen, APAP) is one of the most frequently applied drugs worldwide and is considered generally a safe analgesic and antipyretic drug in therapeutic dosage, which lacks however appreciable anti-inflammatory and antiplatelet activity [10]. The mechanism of the analgesic and antipyretic effects of paracetamol is not fully established, yet inhibition of PGHS activity by paracetamol in different cell and tissue types is generally assumed to be the main mode of paracetamol's analgesic and antipyretic action. PGHS possesses both peroxidase and cyclooxygenase activity. Paracetamol is believed to inhibit the peroxidase catalytic site of PGHS, unlike the majority of nonsteroidal anti-inflammatory drugs (NSAIDs) and the PGHS2 inhibitors. In vitro, paracetamol is a much stronger inhibitor of prostanoid synthesis in endothelial cells than in platelets. In particular, paracetamol is a weak inhibitor of TxA_2_ synthesis in platelets. It is also remarkable that the inhibitory potency of paracetamol is inversely correlated with the PGHS concentration (for a review, see [10]). These particular characteristics distinguish paracetamol from NSAIDs including acetylsalicylic acid (ASA).

In vitro, PGHS activity can be assessed by measuring the production rate of various primary prostanoids, such as PGE_2_, PGI_2,_ and TxA_2_. Because of the remarkable chemical instability of PGI_2_ and TxA_2_, their stable hydrolysis products, that is, 6-keto-PGF_1*α*_ and TxB_2_, respectively, are measured instead of PGI_2_ and TxA_2_ [11]. In vivo, measurement of PGE_2_, 6-keto-PGF_1*α*,_ and TxB_2_ in plasma is associated with artefactual prostanoid synthesis during blood sampling and may lead to incorrect conclusions with regard to PGHS activity [12]. This especially applies to TxA_2_ which is produced in high amounts in activated platelets [13]. Measurement of PGE_2_ in the urine reflects renal PGE_2_ synthesis. By far more reliable is the measurement of major urinary metabolites of prostanoids, such as 2,3-dinor-TxB_2_ for TxA_2_, 2,3-dinor-6-keto-PGF_1*α*_ for PGI_2_, and the major urinary metabolite of PGE_2_ (PGE-MUM) for systemic PGE_2_ production [11]. This can be best accomplished by means of analytical technologies which have high inherent sensitivity and selectivity such as gas chromatography-mass spectrometry (GC-MS) and more so gas chromatography-tandem mass spectrometry (GC-MS/MS) (for a review, see [11]).

Recently, Sudano and colleagues [9] reported that paracetamol (1 g TID for 2 weeks on top of standard cardiovascular therapy) increased ambulatory mean systolic and diastolic blood pressure by about 3 and 2 mmHg, respectively, without changing endothelium and platelet function in patients with coronary artery disease (CAD). Sudano et al. [9] concluded that, particularly in patients at increased cardiovascular risk, use of paracetamol should be evaluated as rigorously as traditional NSAIDs and selective COX2 inhibitors. In that study, plasma and urine PGE_2_ as well as plasma TxB_2_ did not change upon paracetamol administration [9]. However, as mentioned above, measurement of PGE_2_ and TxB_2_ in plasma is prone to artefactual prostanoid synthesis [12, 13], whereas measurement of PGE_2_ in the urine does not provide information about PGHS-catalyzed synthesis of the two antagonists TxA_2_ and PGI_2_ [11].

In humans, oral administration of paracetamol (500 mg) has been reported not to result in decreased excretion rate of 2,3-dinor-TxB_2_, unlike aspirin (500 mg) or indomethacin (50 mg), as measured by GC-MS [14]. Also, in contrast to aspirin (3 g for 2 days), oral administration of paracetamol (3 g for 2 days) has been reported not to reduce urinary excretion of PGE_2_ but to weakly reduce PGE-MUM excretion indicating inhibition of systemic PGE_2_ synthesis [15]. On the other hand, a single oral dose of 500 mg paracetamol has been shown to reduce urinary excretion rate of 2,3-dinor-6-keto-PGF_1*α*_ for 6–8 h by maximally 60% (i.e., inhibition of PGI_2_ synthesis), without reducing urinary excretion rate of 2,3-dinor-TxB_2_ (i.e., no inhibition of TxA_2_ synthesis) [16]. The results of these in vivo studies in human subjects suggest that orally administered paracetamol, at a single dose of 500 mg or at a cumulative dose of 3000 mg per day, does not inhibit remarkably TxA_2_ synthesis, but it may temporarily inhibit PGI_2_ synthesis.

The ramifications between NOS and COX pathways have been frequently investigated in the past (reviewed in [4]), but results are inconsistent. For instance, in murine macrophages, paracetamol, at pharmacologically relevant plasma concentrations (60–120 *μ*M), has been reported not to affect iNOS activity [17]. At suprapharmacological concentrations (2, 5, and 10 mM), paracetamol has been reported to inhibit iNOS gene expression and iNOS activity in RAW 264.7 cell line macrophages [18]. By contrast, paracetamol (up to 10 mM) has been reported not to affect neuronal NOS (nNOS) and iNOS activity in rat cerebellum and HUVECs [19]. Others have reported that paracetamol (100 *μ*M) did not affect nNOS activity in cerebellum but inhibited NOS activity in murine spinal cord slices as measured by the radiolabelled L-citrulline assay [20]. The effect of paracetamol on in vivo in humans is elusive.

Because paracetamol, when applied at pharmacological doses, inhibits the synthesis of the vasodilatatory and antiaggregatory PGI_2_ much stronger and sustainably than the synthesis of the vasoconstrictory and thrombogenic TxA_2_ in humans, we wondered whether the paracetamol-induced shifting of the balance between vasodilatatory/antiaggregatory and vasoconstrictory/thrombogenic COX-related homeostasis may induce processes that lead to enhanced synthesis of the vasodilatatory/antiaggregatory NO, thus counteracting blood pressure fall and platelet activation. Preliminary investigations of our group showed that paracetamol, administered in therapeutic doses to healthy humans (up to 10 mg/kg), did not change whole body NO synthesis (data not shown), suggesting that a potential effect of paracetamol on NOS activity is likely to require much higher, suprapharmacological doses of this drug. In consideration of the toxicological potency of high paracetamol doses, we investigated the effects of a single oral 3 g dose in four healthy volunteers. To our knowledge, the effects of such a high single oral dose of paracetamol on PGI_2_, TxA_2,_ and NO synthesis in humans have not been investigated so far. Because of the high dose used in the human study, paracetamol may induce oxidative stress and decrease NO bioavailability [21]. We therefore measured the oxidative stress biomarker 15(*S*)-8-*iso*-PGF_2*α*_ [22] in plasma and urine. Nitrite in plasma was measured as a biomarker of NO synthesis and bioavailability (reviewed in [23]). In addition, we performed in vitro studies on recombinant endothelial NOS (eNOS) and inducible NOS (iNOS) in rat hepatocytes to test potential effects of paracetamol on NO synthesis and bioavailability.

## 2. Materials and Methods

### 2.1. Subjects and Study Performance

Four healthy nonsmoking male adults (aged 39, 40, 44, and 64 years) participated in the study and gave their informed consent to the study. The volunteers received orally six 500 mg paracetamol tablets (Ratiopharm) at once. Dosage was each 29 mg/kg for volunteer A and volunteer B, 37 mg/kg for volunteer C, and 52 mg/kg for volunteer D. Volunteers were not fasting but they did not eat in the first three hours following paracetamol administration. Before and after paracetamol administration, venous blood and urine were collected in 30 and 60 min intervals over an observation period of 6 h for analysis of biochemical parameters as described below. Venous blood (8 mL) was drawn by using 9 mL EDTA vacutainers (Sarstedt, Germany) and centrifuged immediately (800 ×g, 4°C, 5 min). Plasma was decanted, portioned in 0.1 and 1.0 mL aliquots as required for each biochemical parameter, and stored frozen at −80°C until analysis. Urine from spontaneous micturition was collected in 45 mL polypropylene tubes, aliquoted in 0.1 and 1.0 mL portions according to the requirement of the individual biochemical parameters, and stored at −20°C until analysis.

### 2.2. Analysis of Biochemical Parameters in the Human Study

All samples of this study were analyzed within 10 days after collection. In GC-MS and GC-MS/MS methods, stable-isotope labelled analogs were used as internal standards as reported in the respective references cited below. We found that paracetamol added to pooled human plasma at concentrations of 10, 25, 50, 75, and 100 mg/L did not interfere with the analysis of the biochemical parameters measured in the study plasma samples (data not shown). Data from this study are reported as mean ± standard error of the mean (SEM).

#### 2.2.1. Measurement of Paracetamol

Plasma paracetamol concentration was determined by reverse phase HPLC (250 × 4 mm i.d., 5 *μ*m particle size) with isocratic elution (mobile phase: 45 mM ammonium sulphate-acetonitrile, 10 : 1, v/v; flow rate: 1 mL/min) with UV absorbance detection at 236 nm.

#### 2.2.2. Measurement of Prostanoids and Creatinine

PGI_2_ and TxA_2_ synthesis was assessed by GC-MS/MS by measuring in 1 mL urine aliquots the respective major urinary metabolites [11], that is, 2,3-dinor-6-keto-PGF_1*α*_ and 2,3-dinor-TxB_2_, exactly as described elsewhere [24]. PGE_2_ and free nonconjugated 15(*S*)-8-*iso*-PGF_2*α*_ in urine (1 mL) and free 15(*S*)-8-*iso*-PGF_2*α*_ in plasma (1 mL) were measured by GC-MS/MS after extraction by immunoaffinity column chromatography as described previously [25]. Urinary excretion rate of the eicosanoids was corrected for creatinine excretion [11] and is expressed in nmol prostanoid/mol creatinine. Urine creatinine was measured in 10 *μ*L urine aliquots by GC-MS as reported elsewhere [26].

#### 2.2.3. Analysis of the L-Arginine/NO Pathway

Nitrite and nitrate were measured simultaneously in 100 *μ*L aliquots of plasma or urine by GC-MS as described elsewhere [27]. Urinary excretion rate of nitrite and nitrate was corrected for creatinine excretion as well. Arginine and the endogenous NOS activity inhibitor asymmetric dimethylarginine (ADMA) were measured by GC-MS and GC-MS/MS, respectively, in 100 *μ*L aliquots of ultrafiltrate obtained from plasma by centrifugation according to previously reported procedures [8].

#### 2.2.4. Additional Analyses

Total homocysteine (hCys) in plasma (0.1 mL) was measured by a commercially available fluorescence polarimetry immunoassay (FPIA). In vivo CYP activity [28] was assessed by measuring oleic acid oxide (*cis*-EpOA) in 1 mL aliquots of plasma as described elsewhere [29].

#### 2.2.5. Quality Control

Quality control (QC) samples were analyzed alongside study samples for all biochemical parameters. Accuracy and precision in the QC samples were within generally accepted ranges; that is, bias and imprecision levels were below 20%.

### 2.3. Effect of Paracetamol on Recombinant Human eNOS Activity

The effect of paracetamol on NOS activity in vitro was investigated by using a commercially available (ALEXIS, Grünberg, Germany) recombinant human endothelial NOS (heNOS) and by measuring simultaneously formation of [^15^N]nitrite and [^15^N]nitrate from L-[*guanidine-* ^15^N_2_]arginine by means of a GC-MS assay [30]. Incubations were performed at 37°C in 50 mM potassium phosphate buffer (1000 *μ*L, pH 7) containing heNOS (50 *μ*g/mL), L-[*guanidine*-^15^N_2_]-arginine (20 *μ*M, Cambridge Isotope Labs, Andover, MA, USA), and all NOS cofactors (all purchased from Sigma-Aldrich, Steinheim, Germany) and prosthetic groups (10 *μ*M tetrahydrobiopterin, 800 *μ*M NADPH, 5 *μ*M FAD, 5 *μ*M for FMN, 500 nM calmodulin, and 500 *μ*M CaCl_2_ (Merck, Darmstadt, Germany)). Reactions were terminated by addition of 400 *μ*L aliquots of ice cold acetone and samples were processed for GC-MS analysis of [^15^N]nitrite and [^15^N]nitrate. Unlabeled nitrite and nitrate were used as internal standards for [^15^N]nitrite and [^15^N]nitrate, respectively. Data are shown as mean ± standard deviation (SD) from two independent experiments.

### 2.4. Effect of Paracetamol on iNOS Activity in Cultured Rat Hepatocytes Proliferating In Vitro

The effect of paracetamol on iNOS activity was investigated in primary rat hepatocytes proliferating in vitro as described recently by measuring formation of [^15^N]nitrite and [^15^N]nitrate by GC-MS [31]. In some experiments, LiCl (10 mM) was used to enhance expression of iNOS-mRNA and cell growth [31]. Incubations were performed at 37°C in the presence of 5 mM L-[*guanidine*-^15^N_2_]-arginine added at the time -22 h. Reactions were terminated by addition of 400 *μ*L aliquots of ice cold acetone, and samples were processed for GC-MS analysis of [^15^N]nitrite and [^15^N]nitrate. Data are shown as mean ± SD from three independent experiments.

### 2.5. Statistical Analysis

Because of considerable differences in the baseline concentrations of some of the biochemical parameters measured in the four subjects, changes and statistical significance were calculated by setting the respective baseline levels to 100%. Statistical significance (*P* < 0.05) was evaluated by using unpaired *t*-test and comparing the data obtained at various times to the baseline values or to the 0.5 h values when percentage changes were compared.

## 3. Results 

### 3.1. Effect of High-Dose Paracetamol on COX, NOS, and CYP Activity and on Oxidative Stress in Humans

**Figure 1:**
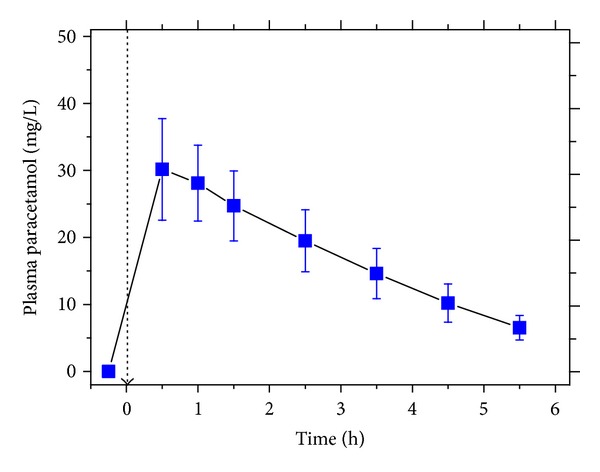
Plasma concentration of paracetamol before and after administration (time zero is indicated by the dashed arrow) of a single oral 3 g dose of paracetamol to four male subjects. Data are shown as mean ± SEM.

Mean maximum paracetamol plasma concentration (*C*_max⁡_) was 30.2 mg/L (200 *μ*mol/L) (Figure 1). This value is consistent with a *C*_max⁡_ value of about 20 mg/L that has been reached after oral administration of 2000 mg of paracetamol [32]. In the urine samples, we measured by reverse phase HPLC with UV absorbance detection comparable creatinine-corrected excretion rates of the paracetamol glucuronide and sulphate metabolites (data not shown).

**Figure 2:**
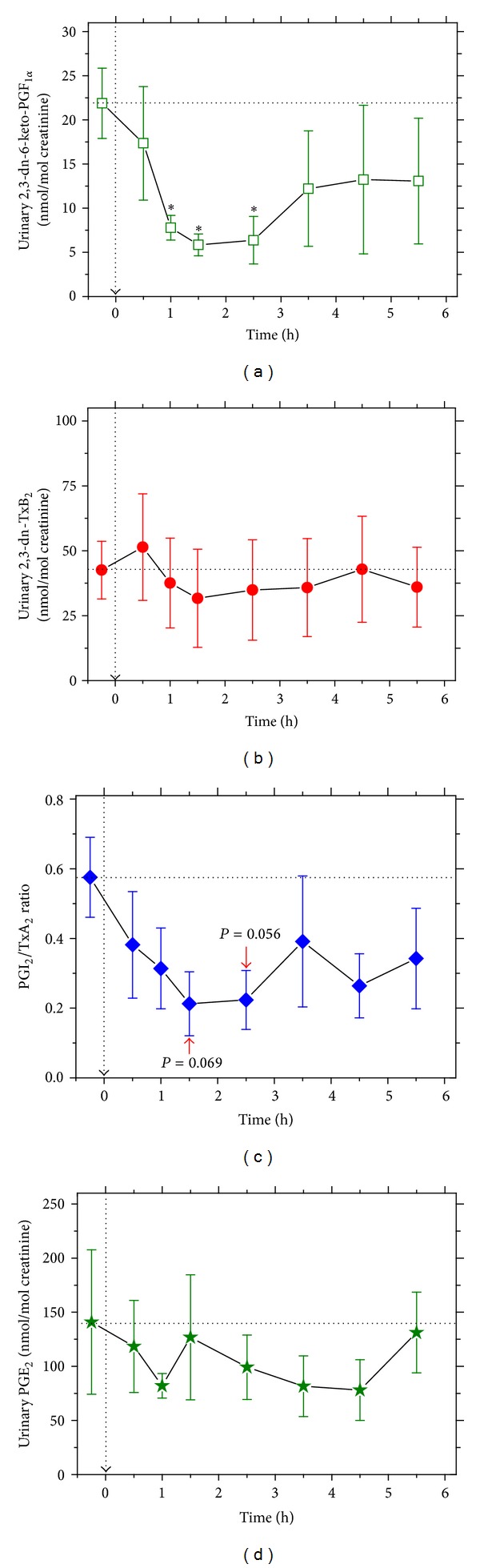
Effect of a single oral 3 g dose of paracetamol on systemic prostacyclin and thromboxane synthesis and on renal synthesis of PGE_2_ in four healthy volunteers (time zero and baseline values are indicated by dashed arrows). (a) Creatinine-corrected urinary excretion of 2,3-dinor-6-keto-prostaglandin F_1*α*_ (2,3-dn-6k-PGF_1*α*_) as a measure of systemic PGI_2_ synthesis. (b) Creatinine-corrected urinary excretion of 2,3-dinor-thromboxane B_2_ (2,3-dn-TxB_2_) as a measure of systemic TxA_2_ synthesis. (c) PGI_2_/TxA_2_ molar ratio calculated from the 2,3-dn-6k-PGF_1*α*_ and 2,3-dn-TxB_2_ excretion rates shown in (a) and (b), respectively. (d) Creatinine-corrected urinary excretion of PGE_2_ as a measure of renal PGE_2_ synthesis. An asterisk in (a) indicates statistical significance (*P* < 0.05) compared to basal values. Data are shown as mean ± SEM.

Upon 3 g paracetamol intake, considerable and sustained decrease in creatinine-corrected 2,3-dinor-6-keto-PGF_1*α*_ excretion rate was seen, suggesting strong PGI_2_ inhibition by paracetamol (Figure 2(a)). Maximum and statistically significant PGI_2_ inhibition of about 60 to 70% was reached 1 h, 1.5 h, and 2.5 h after paracetamol administration. The extent of the decrease seen in the 2,3-dinor-6-keto-PGF_1*α*_ excretion rate in the present study is comparable to that seen upon administration of a 500 mg oral paracetamol dose [14]. Paracetamol caused only a moderate, statistically insignificant decrease in 2,3-dinor-TxB_2_ excretion in the four volunteers (Figure 2(b)). In three out of the four volunteers, maximum TxA_2_ inhibition of about 70% was reached 1.5 h after administration, but the duration of TxA_2_ inhibition was relatively short (not shown).  Figure 2(c) shows that the PGI_2_/TxA_2_ molar ratio decreased by a factor of 2 to 3 upon paracetamol administration, although statistical significance failed by a hair 1.5 h (*P* = 0.069) and 2.5 h (*P* = 0.056) after paracetamol ingestion. Paracetamol seemed to decrease very weakly the excretion of PGE_2_ in the urine (Figure 2(d)), suggesting that even 3 g of paracetamol taken at once is not able to inhibit renal synthesis of PGE_2_ in the four volunteers enrolled in the study.

**Figure 3:**
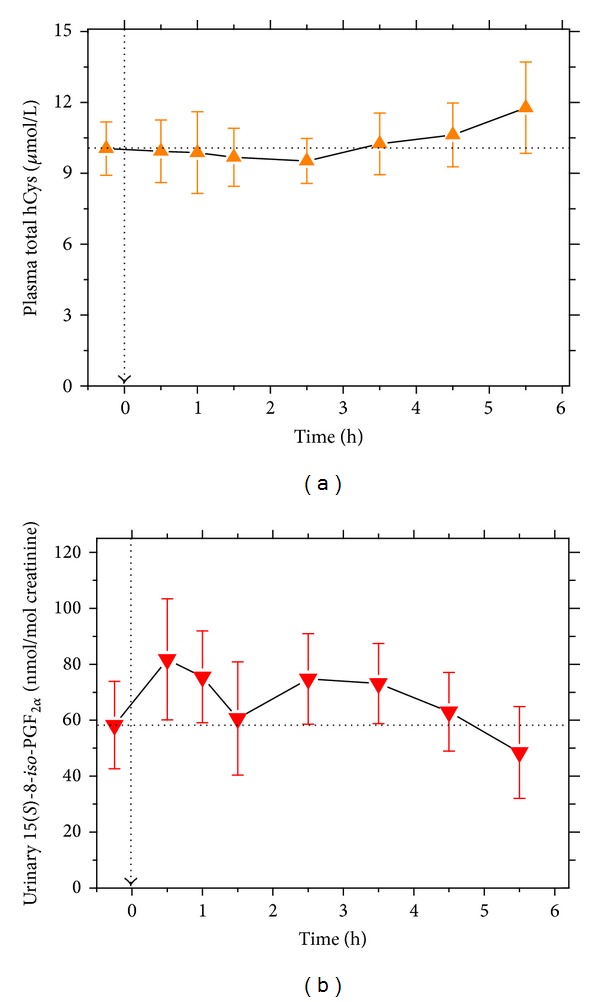
Effect of a 3 g single oral dose of paracetamol on oxidative stress in four healthy volunteers (time zero and baseline value are indicated by dashed arrows). (a) Plasma total homocysteine (hCys) concentration and (b) creatinine-corrected urinary excretion rate of 15(*S*)-8-*iso*-prostaglandin F_2*α*_ (15(*S*)-8-*iso*-PGF_2*α*_) as a measure of oxidative stress. Data are shown as mean ± SEM.

Paracetamol-induced changes in systemic prostacyclin synthesis were not accompanied by noteworthy changes in the plasma concentration of total hCys (Figure 3(a)) or in the urinary excretion rate of free 15(*S*)-8-*iso*-PGF_2*α*_ (Figure 3(b)), suggesting no elevation or reduction of oxidative stress upon high-dosed paracetamol administration.

**Figure 4:**
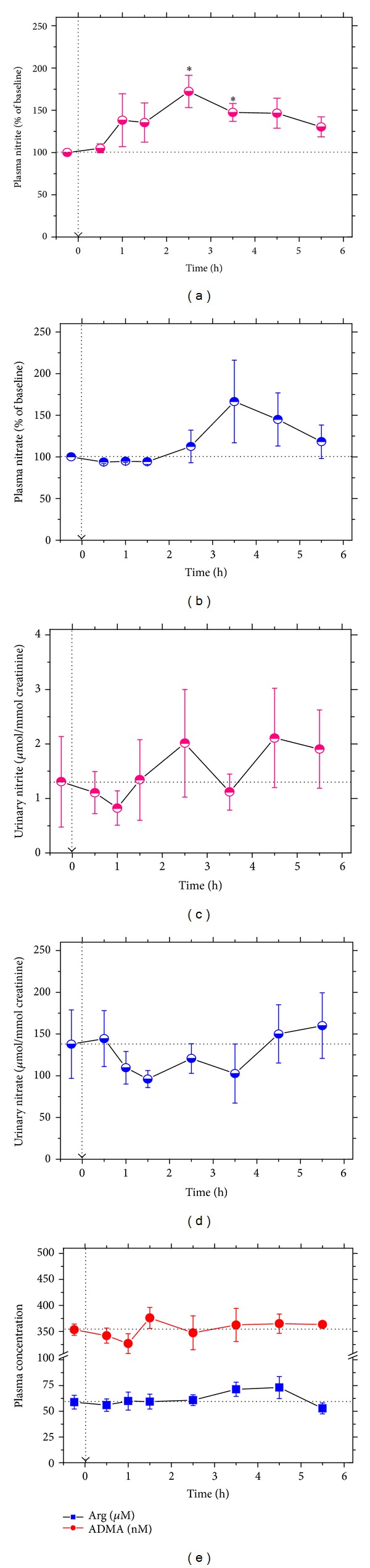
Effect of a 3 g single oral dose of paracetamol on plasma (a) and urine (c) nitrite, plasma (b) and urine (d) nitrate, and plasma arginine (Arg) and asymmetric dimethylarginine (ADMA) (e) in four healthy volunteers (time zero and baseline values are indicated by dashed lines). Data in plasma are shown as percentage changes of the baseline plasma nitrite concentrations (1.26, 3.04, 3.76, and 4.05 *μ*M) and baseline plasma nitrate concentrations (37.3, 42.5, 26.8, and 52.9 *μ*M), respectively. Data are shown as mean ± SEM. An asterisk indicates statistical significance (*P* < 0.05) compared to the 0.5 h values.

Because of the considerable difference in the baseline plasma nitrite and nitrate concentrations measured in the four subjects, changes in plasma nitrite and nitrate were calculated and presented as percentage of the respective baseline levels.  Figure 4(a) shows moderate increases in plasma nitrite concentration which were statistically significantly higher when the 2.5 and 3.5 h values were compared with the 0.5 h values. Changes in plasma nitrate concentrations (Figure 4(b)) and urine nitrite (Figure 4(c)) and urine nitrate (Figure 4(d)) excretion were not statistically significantly different. Finally, plasma Arg and ADMA concentrations did not change upon paracetamol ingestion (Figure 4(e)).

**Figure 5:**
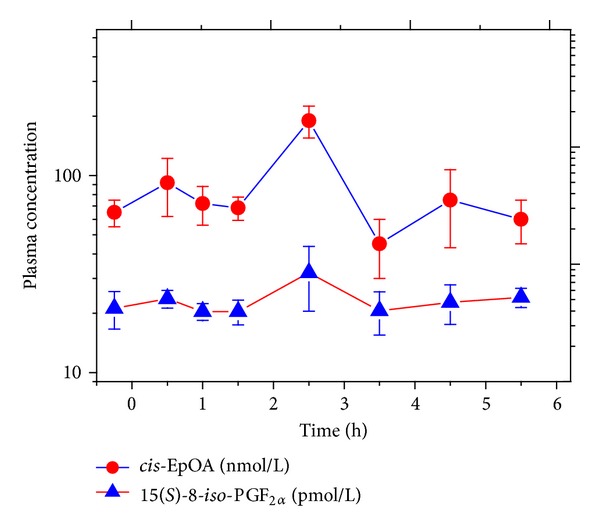
Effect of a 3 g single oral dose of paracetamol on serum* cis*-epoxyoctadecanoic acid (*cis*-EpOA) and 15(*S*)-8-*iso*-PGF_2*α*_ in four healthy volunteers (time zero and baseline value are indicated by dashed lines). Data are shown as mean ± SEM. Note the logarithmic scale on the *y*-axis. Only the 2.5 h concentration of* cis*-EpOA was statistically significantly different (*P* < 0.05) compared to the baseline.

Previously, we showed that the whole body activity of CYP isoforms can be assessed by measuring the concentration of the free, that is, nonesterified, oleic acid oxide* cis*-EpOA in plasma [28].  Figure 5 shows an abrupt increase in mean plasma* cis*-EpOA concentration 2.5 h after paracetamol administration followed by an abrupt fall to baseline level 1 h later. This finding may suggest a very short-term paracetamol-induced elevation of CYP activity. However, we also found a very similar change in the plasma concentration of free 15(*S*)-8-*iso*-PGF_2*α*_ (Figure 5). Previously, we observed that addition of phospholipase A_2_ (PLA_2_) to human serum increased in parallel the concentration of both free* cis*-EpOA and free 15(*S*)-8-*iso*-PGF_2*α*_ [25]. Therefore, the temporary short-time increases in* cis*-EpOA and 15(*S*)-8-*iso*-PGF_2*α*_ seen 2.5 h after paracetamol administration may have resulted from release of presumably hepatic PLA_2_ into the blood.

### 3.2. Effects of Paracetamol on Recombinant heNOS Activity and iNOS in Rat Hepatocytes

**Figure 6:**
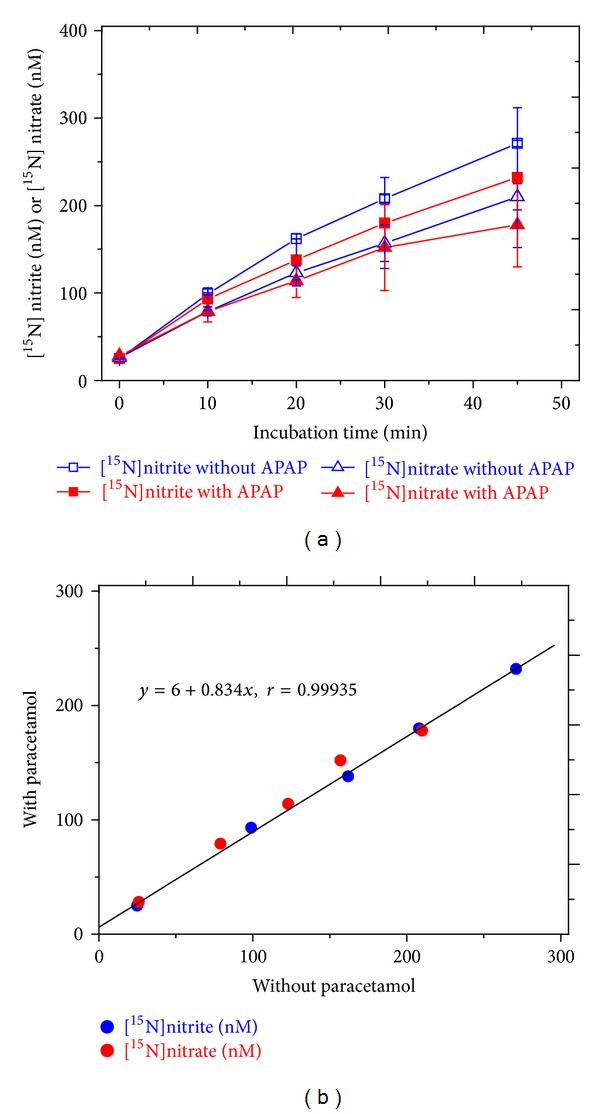
Effect of paracetamol (APAP) at 100 *μ*M (15 mg/L) on the formation of [^15^N]nitrite and [^15^N]nitrate in an incubation mixture of recombinant heNOS upon incubation time (a) and linear regression analysis between [^15^N]nitrite and [^15^N]nitrate concentrations measured in the presence and absence of paracetamol (b). Data in (a) are shown as mean ± SD from two independent experiments; no statistical analysis was performed.

**Figure 7:**
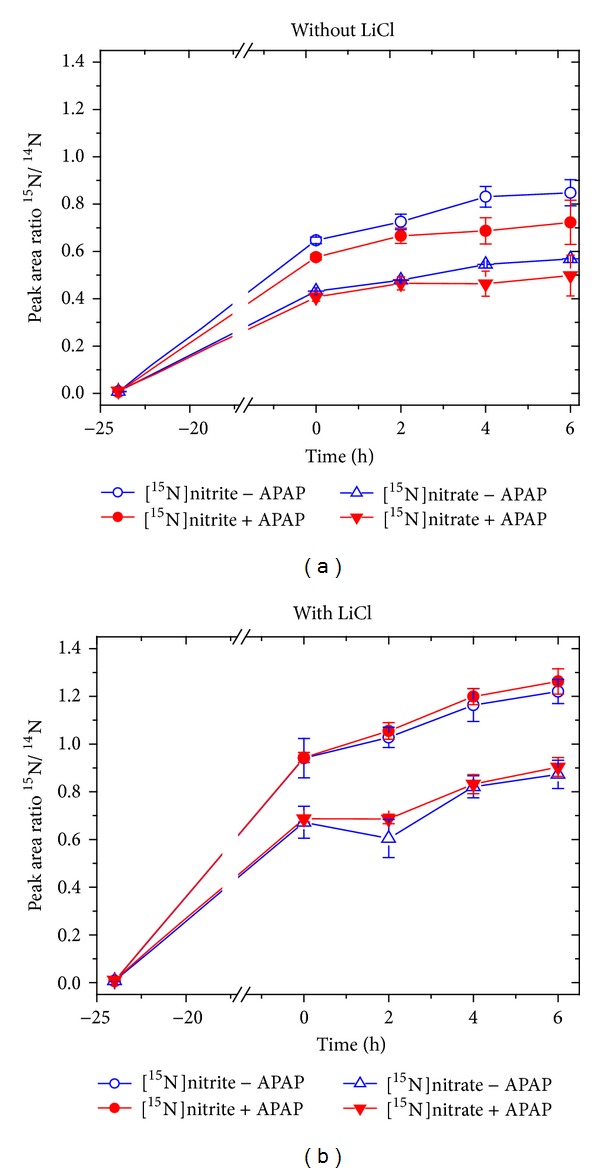
Effect of paracetamol (APAP) at 100 *μ*M (15 mg/L) on the peak area ratio of m/z 47 for [^15^N]nitrite to m/z 46 for [^14^N]nitrite (a) and on the peak area ratio of m/z 63 for [^15^N]nitrate to m/z 62 for [^14^N]nitrate (b) upon incubation of adult rat hepatocytes with L-[*guanidine*-^15^N_2_]-arginine (5 mM) in the absence and in the presence of LiCl (1 mM) for the indicated times at 37°C as described elsewhere [8]. Reactions were terminated by addition of 400 *μ*L aliquots of ice cold acetone and samples were further processed for GC-MS analysis. Data are shown as mean ± SD from three independent experiments; no statistical analysis was performed.

At the therapeutically relevant concentration of 100 *μ*M (i.e., 15 mg/L), paracetamol had only a weak effect on the formation of [^15^N]nitrite and [^15^N]nitrate in incubation mixtures of a recombinant heNOS (Figure 6). Linear regression of analysis between the concentrations of [^15^N]nitrite and [^15^N]nitrate in the presence of paracetamol versus the concentrations of [^15^N]nitrite and [^15^N]nitrate in the absence of paracetamol revealed a slope value of 0.834 (Figure 6(b)). This finding suggests that paracetamol inhibited heNOS-catalyzed ^15^NO formation (i.e., [^15^N]nitrite and [^15^N]nitrate) from L-[*guanidine*-^15^N_2_]-arginine in average by 16.6%, notably for incubation times longer than 10 min. Similar small effects of paracetamol on iNOS were also seen in experiments with adult rat hepatocytes proliferating in vitro independent of the presence of LiCl (Figure 7). It is well established that peroxynitrite can nitrate paracetamol to 3-nitroparacetamol [33]. In the paracetamol-containing samples from both the recombinant heNOS and the rat hepatocytes iNOS, no 3-[^15^N]nitroparacetamol was detected by GC-MS/MS above the limit of quantitation (about 1 nM), suggesting no formation of peroxynitrite (data not shown).

## 4. Discussion

### 4.1. General Remarks and Aim of the Study

Paracetamol is generally assumed to increase oxidative stress and is therefore commonly used in animal models of oxidative stress, in which paracetamol is administered in exorbitant high doses [21]. Whether paracetamol, when administered at therapeutic doses, also acts as a prooxidant is unknown. Paracetamol is known to interact with many enzymes such as CYP, COX, and NOS, which themselves are known to contribute to oxidative stress, for instance, by producing superoxide radical anions. While the inhibitory effect of paracetamol on prostacyclin synthesis in vivo in humans is well established [16], its effects on thromboxane and NO synthesis as well as on CYP activity are incompletely understood. This may be due to insufficiently high intracellular paracetamol concentrations when this drug is administered in therapeutic doses, for instance, by oral administration of a 500 mg paracetamol tablet. The aim of the present work was to investigate in healthy humans the effects of high-dosed paracetamol (i.e., 3 g) on the activity of the COX, NOS, and CYP, as well as on oxidative stress. Given the well-known hepatotoxicity of paracetamol, only four healthy subjects were enrolled in the human study. By using paracetamol concentrations that are expected to prevail for a considerable period of time after administration of a single 3 g oral dose to humans, we investigated the effects of paracetamol at suprapharmacological concentrations on the activity of two NOS isoforms in vitro, that is, on recombinant human eNOS and iNOS in rat hepatocytes.

### 4.2. Effects of Paracetamol on the Cyclooxygenase Pathway

Considering a mean fraction (oral bioavailability, *F*) value of 88% for paracetamol [32], its mean distribution volume (*V*_*D*_) in the volunteers of the human study described in this paper is estimated to be 88 L. This value is almost 25 times higher than the estimated volunteers' plasma volume and suggests that paracetamol may reach concentrations up to about 5000 *μ*M in other body compartments except for red blood cells. Such high concentrations would be high enough to inhibit prostacyclin (PGI_2_) and thromboxane (TxA_2_) synthesis in endothelial cells and platelets, respectively [10].

Indeed, paracetamol, at the high single oral dose of 3 g, potently inhibited PGHS-catalyzed synthesis of PGI_2_, a potent vasodilator and inhibitor of platelet aggregation. By contrast, the synthesis of TxA_2_, a potent vasoconstrictor and platelet activator, was found not to be significantly inhibited by paracetamol in four subjects. Relative effects of drugs on PGI_2_ and TxA_2_ synthesis are commonly estimated by using the molar ratio of the prostanoids [34]. In our study, oral administration of a single 3 g oral paracetamol dose decreased the average PGI_2_/TxA_2_ molar ratio from about 0.6 before administration to values ranging between 0.4 and 0.2 after administration, thus shifting the vasodilatatory (PGI_2_)/vasoconstrictory (TxA_2_) balance at the cost of vasodilatation. A consequence of such a shift may be an increase of blood pressure. Indeed, Sudano and colleagues found that chronic administration of paracetamol to CAD patients at a lower dose (1 g TID for 2 weeks) than in the present study resulted in small blood pressure increase [9]. It is worth mentioning that in the study by Sudano et al. [9] considerably lower plasma paracetamol concentrations had been reached in comparison to those we measured in our study. In confirmation of previous studies [9, 15], we found that excretion of PGE_2_ did not change upon paracetamol administration, suggesting that even a high dose of 3 g paracetamol did not alter significantly renal PGE_2_ production in the volunteers.

### 4.3. Effects of Paracetamol on the L-Arginine/NO Pathway

The effects of paracetamol on NOS expression and activity have been studied by several groups. Yet, the observations are contradictory [17–20]. In our human study, paracetamol increased temporarily the concentration of nitrite in plasma. As the major fraction of circulating nitrite may originate from NO produced in the endothelium [23], our in vivo results may indicate that paracetamol increased eNOS activity and/or eNOS expression 2.5 to 3.5 h after administration. Yet, alternative ways such as paracetamol-induced reduction of nitrate to nitrite may have also increased plasma nitrite concentrations in the volunteers. Paracetamol did not change the plasma concentration of two other main parameters of the L-arginine/NO pathway, that is, L-arginine and ADMA. In vitro, paracetamol had only a very weak inhibitory effect on isolated recombinant heNOS and on iNOS in adult rat hepatocytes proliferating in vitro. LiCl that is known to induce expression and activity of iNOS in rat hepatocytes [31] increased iNOS activity but did not alter NO bioavailability. Thus, neither paracetamol nor LiCl influenced iNOS-related oxidative stress in rat hepatocytes.

### 4.4. Effects of Paracetamol on the Cytochrome P450 Pathway

Paracetamol is oxidized by the CYP family to NAPQI (*N*-acetyl-*p*-benzoquinone imine), the toxic intermediate of paracetamol. Unsaturated fatty acids including arachidonic acid and oleic acid are substrates for CYP enzymes [28, 35], and some of the arachidonic acid epoxides are vasoactive compounds [35]. At high concentrations (e.g., 1000 *μ*M), paracetamol may inhibit the activity of CYP isoforms. In our human study, paracetamol increased temporarily the plasma concentration of the oleic acid oxide* cis*-EpOA. As* cis*-EpOA is a marker of CYP activity in humans [28], this finding may suggest that paracetamol increases CYP activity for a very short period of time. Another explanation for the very short-lasting increase in plasma* cis*-EpOA concentration could be activation of extracellular phospholipase A_2_ (PLA_2_) activity or release of hepatic PLA_2_ into the blood stream by paracetamol, because a considerable fraction of* cis*-EpOA is found esterified to human serum lipids [28]. The latter explanation is supported by the finding that the plasma concentration of free 15(*S*)-8-*iso*-PGF_2*α*_ displayed a similar course including the sharp maximum like* cis*-EpOA in the present human study. It is worth mentioning that both 15(*S*)-8-*iso*-PGF_2*α*_ and* cis*-EpOA are released in parallel from serum lipids upon incubation with PLA_2_ [28]. At present, very little is known about paracetamol effects on PLA_2_ activity and/or expression. In contrast to indomethacin, paracetamol (at 1000 *μ*M) was found not to inhibit extracellular PLA_2_ activity as measured using radiolabelled oleic acid esterified to* E. coli* membranes [36]. In mice, hepatotoxicity induced by paracetamol at a dose of 400 mg/kg, that is, about 10 times higher than in our human study, was found to be associated with a time-dependent mode with increased secretion of hepatic PLA_2_ which was exacerbated in the absence of hepatic COX-2 [37]. Thus, the temporary increase in* cis*-EpOA and 15(*S*)-8-*iso*-PGF_2*α*_ observed in our study may be due to paracetamol-induced short-term hepatotoxicity in the healthy subjects.

### 4.5. Effects of Paracetamol on Oxidative Stress

In the human study, paracetamol (3 g) did not increase oxidative stress as assessed by measuring urinary excretion of the oxidative stress biomarker 15(*S*)-8-*iso*-PGF_2*α*_ [21, 22]. As discussed above, the sharp and short-lasting increase in the plasma concentration of free 15(*S*)-8-*iso*-PGF_2*α*_ is likely to be due to temporary release of PLA_2_ from the liver and/or due to activation of extracellular PLA_2_. At this high dose, paracetamol did not increase plasma total hCys which is generally assumed to be associated with oxidative stress. Given the ROS-scavenging phenolic moiety of paracetamol (*N*-acetyl-*p*-aminophenol), the failure of paracetamol to enhance oxidative stress seems reasonable. The F_2_-isoprostane 15(*S*)-8-*iso*-PGF_2*α*_ is known to be produced from AA by the catalytical action of COX [38]. In contrast to acetylsalicylic acid, indomethacin, and celecoxib [25, 39], our study indicates that paracetamol (3 g) does not inhibit COX-dependent formation of 15(*S*)-8-*iso*-PGF_2*α*_ in humans.

## 5. Conclusion

**Figure 8:**
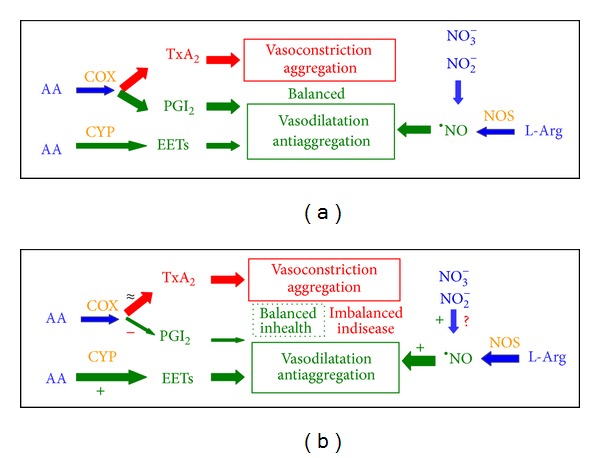
Simplified scheme showing the proposed contribution of three major enzymatic pathways to the vasoconstriction, vasodilatation, and aggregation in the vasculature without (a) and with (b) administration of paracetamol (acetaminophen) in health and disease. In platelets, arachidonic acid (AA) is converted by COX-1 to the vasoconstrictor and aggregator TxA_2_. In endothelial cells, AA is converted by COX-2 to PGI_2_, and L-Arg is oxidized by NOS to ^*∙*^NO; PGI_2_ and ^*∙*^NO are both vasodilatators and antiaggregators. AA is converted by CYP epoxygenases (CYP) to the vasodilatators epoxyeicosatrienoic acids (EETs). (a) In the absence of COX inhibitors including paracetamol and in health, production of TxA_2_, PGI_2_, ^*∙*^NO, and EETs guarantees a balance between vasoconstriction/aggregation and vasodilatation/antiaggregation. (b) Paracetamol and other COX inhibitors shift this balance in favour of COX-dependent vasoconstriction/aggregation. In response to this shift, ^*∙*^NO and EETs formation is increased in order to compensate the imbalance. In health, this compensation succeeds and blood pressure and platelet aggregation do not change. In endothelium dysfunction-related diseases, such as coronary artery disease (CAD), this compensation is insufficient and leads to moderate increase in blood pressure [9]. –, +, and *≈* mean inhibition, activation, and no remarkable change, respectively. The thickness of the arrows is quantitative but not true to scale measure of the contribution of the individual pathways and paracetamol.

We investigated in vitro and in vivo effects of paracetamol, an analgesic and antipyretic phenolic drug, on the L-Arg/NO, AA/COX, and CYP biochemical pathways and on oxidative stress. At the high single oral dose of 3 g, paracetamol did not alter oxidative stress in vivo. At suprapharmacological concentrations, paracetamol also did not alter oxidative stress in vitro as revealed by the unchanged nitrite-to-nitrate molar ratios measured in incubation mixtures of recombinant heNOS and in cultures of adult rat hepatocytes that express iNOS. The potent PGI_2_ inhibition by high-dosed paracetamol in the healthy subjects of the present study suggests that the relatively small increase in blood pressure seen in CAD patients by others [9] is likely to be due to compensatory mechanisms that involve enhanced formation of vasodilators. Potential candidates are NO and epoxyeicosatrienoic acids (EETs). In the circulation, NO can be produced from L-arginine by the catalytic action of eNOS and/or from nitrite/nitrate. EETs are produced from arachidonic acid by the catalytic action of the CYP family [34]. Our results suggest that in healthy subjects NO may compensate the loss of the vasodilatory and antiaggregatory prostacyclin caused by high-dosed paracetamol (Figure 8). Also, paracetamol does not increase oxidative stress even when given at suprapharmacological doses. We assume that in healthy humans the paracetamol-induced shift of the PGI_2_/TxA_2_ balance is counteracted by concomitant increase in circulating NO production. The underlying mechanisms remain elusive. Possible contributing mechanisms may include elevation of NO in the endothelium and conversion of nitrate to nitrite and its consecutive reduction to NO. In patients suffering from cardiovascular diseases, that is, with dysfunctional endothelium, paracetamol only partially counteracts its unfavorable vasodilatory/vasoconstrictory effect via NO.

## Abbreviations

AA: Arachidonic acid
ADMA: Asymmetric dimethylarginine
APAP: Acetaminophen (i.e., paracetamol)
Arg: Arginine
ASA: Acetylsalicylic acid
CAD: Coronary artery disease
CYP: Cytochrome P450
COX: Cyclooxygenase
EETs: Epoxyeicosatrienoic acids
FPIA: Fluorescence polarimetry immunoassay
GC-MS: Gas chromatography-mass spectrometry
GC-MS/MS: Gas chromatography-tandem mass spectrometry
HUVECs: Human umbilical vein endothelial cells
tHyc: Total homocysteine
NO: Nitric oxide
NOS: Nitric oxide synthase
eNOS: Endothelial nitric oxide synthase
heNOS: Human endothelial nitric oxide synthase
iNOS: Inducible nitric oxide synthase
nNOS: Neuronal nitric oxide synthase
NSAIDs: Nonsteroidal anti-inflammatory drugs
PG: Prostaglandin
PGE_2_: Prostaglandin E_2_
PGE-MUM: Prostaglandin E-major urinary metabolite
PGHS: Prostaglandin H synthase
PGI_2_: Prostacyclin
PLA_2_: Phospholipase A_2_
QC: Quality control
ROS: Reactive oxygen species
SD: Standard deviation
SEM: Standard error of the mean
TID: ter in di (three times a day)
TxA_2_: Thromboxane A_2_.
